# Improving the quality of malaria diagnosis in southern Africa through the development of a regional malaria slide bank

**DOI:** 10.1186/s12936-021-03899-5

**Published:** 2021-09-08

**Authors:** Bhavani Moodley, Anderson Chinorumba, Cheryl Hamman, Avhatakali Matamba, Chadwick H. Sikaala, Immo Kleinschmidt, John Frean

**Affiliations:** 1 Centre for Emerging Zoonotic and Parasitic Infections, National Institute for Communicable Diseases, Johannesburg, South Africa; 2 World Health Organization, Harare, Zimbabwe; 3 SADC Malaria Elimination Eight Secretariat, Windhoek, Namibia; 4 Wits Research Institute for Malaria, School of Pathology, Faculty of Health Sciences, University of the Witwatersrand, Johannesburg, South Africa; 5 MRC International Statistics and Epidemiology Group, Department of Infectious Disease Epidemiology, London School of Hygiene & Tropical Medicine, London, UK

**Keywords:** Malaria slide bank, Quality assurance, Malaria microscopy, Proficiency testing

## Abstract

**Background:**

A malaria slide bank (MSB) is a useful asset for any malaria microscopy testing laboratory to have access to. However, it is not feasible for every country to have its own MSB. If countries are able to pool their resources, a regional MSB is a viable solution. This paper describes the methodology, costing and lessons learnt of establishing and maintaining an MSB over a 3-year period, for a Southern Africa Development Community region.

**Methods:**

A national reference laboratory in South Africa was granted funding for setting up the MSB; it possessed experienced staff and suitable resources. Two additional full-time personnel were employed to carry out the activities of this project. Strict protocols for donor/patient blood sample screening, smear preparation, mass staining, quality control and slide validation were followed. Slides from the MSB were used for training and proficiency testing purposes. The initial and recurrent yearly costs to set up and maintain the MSB were calculated.

**Results:**

Over 35 months, 154 batches (26,623 slides) were prepared; the majority were *Plasmodium falciparum*. Ninety-two percent (141/154) of batches passed internal quality control, and 89% (93/104) passed external validation. From these slides, two training slide sets and six proficiency testing slide sets were sent out. The initial year’s cost to establish an MSB was calculated at approximately $165,000, and the recurrent year-on-year cost was $130,000.

**Conclusions:**

The key components for maintaining a high-quality MSB are consistent funding, competent staff and adherence to standardized protocols. Travel to malaria-endemic areas for access to non-falciparum malaria species, and dilution of *P. falciparum* blood to desired parasite densities, are extremely useful to ensure variety. The MSB created here supported multiple laboratories in eight countries, and has the potential to expand.

**Supplementary Information:**

The online version contains supplementary material available at 10.1186/s12936-021-03899-5.

## Background

Population mobility across borders, where it involves movement of malaria parasite-infected people, poses challenges to countries’ efforts to achieve malaria elimination [1–3]. Joint cross-border efforts form a key part of the framework to eliminate malaria from endemic regions worldwide [2, 4]. The Southern Africa Development Community (SADC) Malaria Elimination Eight (E8) was established by SADC heads of state in 2009, to coordinate and execute regional strategies with the aim of eliminating malaria by 2030 [2]. The eight countries are Angola, Botswana, Eswatini, Mozambique, Namibia, South Africa, Zambia and Zimbabwe.

Universal access to malaria diagnosis is part of the World Health Organization (WHO) strategic framework to move countries towards malaria elimination [5, 6]. Quality-assured diagnosis, either microscopy or rapid diagnostic tests (RDTs), is recommended before treatment is administered to patients and is an essential requirement for the certification of malaria-free status by the WHO [6]. In 2017, the E8, through a grant from the Global Fund against Aids, Tuberculosis and Malaria, and with support from the WHO Regional Office for Africa (AFRO), initiated activities that were designed to build and strengthen high-quality diagnostic capacity in the region. These were: (1) the development of a regional malaria slide bank (MSB); (2) the formal assessment of core microscopists; and (3) the training of core microscopists to become proficient malaria microscopy trainers. These activities are part of the WHO’s 10 key components of a quality assurance (QA) system in malaria microscopy diagnosis [7].

A collection of well-characterized, good quality blood films is an indispensable asset to a malaria QA system. The objectives of an MSB are to provide sets of known, replicate slides for training, external and internal assessment of microscopists, and for proficiency testing schemes (PTS). The provision of such a resource at regional level is likely to be highly cost-effective compared to duplication of such facilities in each country. Furthermore, most countries lack the infrastructure, expertise and resources to establish an MSB.

This paper details the process and cost of setting-up and maintaining a regional MSB. In 2018, the National Institute for Communicable Diseases (NICD) was awarded an E8-administered grant to establish an MSB. The NICD is a division of the South African Department of Health’s National Health Laboratory Service, and provides reference diagnostic laboratory services for the country and region, as well as training and research functions for communicable diseases of public health importance [8]. As such, the NICD performs the role of a national malaria reference laboratory. The NICD maintains ISO 15189 accreditation and was also previously ISO 17043-accredited in its role as an EQA provider.

## Methods

The WHO Malaria Microscopy QA Manual (Chapter 12) was used for guidance on the requirements of an MSB and the steps involved to develop it [7]. Using this manual, in addition to its own experience and procedures, the NICD went about developing an MSB for the E8 region. In May 2018, the NICD employed two medical technologists who were dedicated to this project, following thorough internal training on all processes. They attended an MSB training workshop organized and funded by the WHO AFRO in August 2018. Among the facilitators were staff from the Research Institute for Tropical Medicine (RITM), Philippines. The RITM has the only WHO-approved MSB in the world and graciously shared its methods. See Additional file 1 for details of staff training.

The aim was to prepare 50 batches of at least 100 slides each in year 1, and 100 batches each in year 2 and 3. Here, a batch is defined as all replicate slides prepared together from the same blood tube. An MSB ideally should acquire blood smears of all human species of *Plasmodium* and malaria-negative smears, as well as non-malaria parasites such as trypanosomes and microfilariae. Slide preparation began immediately after staff were employed and trained. As the NICD is not a routine testing facility, nearby routine diagnostic laboratories around Johannesburg (see Additional file 2) were requested to kindly notify the NICD when they had a positive malaria sample. After the EDTA blood sample was used for diagnostic purposes, the residual sample, usually between 1 and 4 ml, was fetched. Malaria-negative blood was collected from healthy staff who volunteered to donate 4–8 ml of their blood. Ethical clearance was obtained from the University of Witwatersrand Human Research Ethics Committee (clearance certificate number M161061) for the use of samples collected from patients with parasitic infections, and the collection of samples from healthy donors for MSB purposes.

The quality of samples was assessed by microscopically examining the parasites and blood components. If acceptable, mass blood smear preparation was carried out. Smears were left to dry for at least 24 h, after which thin films were fixed with 100% methanol (Merck KGaA, 64271 Darmstadt, Germany). Mass staining was performed for 30 min with a 3% Giemsa solution (Merck KGaA, 64271 Darmstadt, Germany), prepared with a phosphate buffer of pH 7.2 (Diagnostic Media Products, Johannesburg, South Africa). Desired parasite densities (parasitaemias) were produced by diluting positive blood with healthy donor blood of the same ABO blood group. After staining, slides underwent internal quality control (QC), whereby slides were macroscopically and microscopically examined, and parasite counts were performed on all malaria-positive slides [9]. Poor quality slides failed QC and were discarded. Slides from batches that passed QC were sent to WHO-certified level 1 microscopists from within the E8 region, for validation. Batches that passed validation were added to the MSB inventory. For long-term storage, slides were coverslipped using an automated coverslipping instrument (Leica CV5030, Leica Biosystems, Buffalo Grove, IL 60089 United States) and labelled before being stored in slide cabinets. A real-time PCR assay using a commercial kit (RealStar Malaria Screen & Type PCR Kit 1.0, Altona Diagnostics, Hamburg, Germany; QuantStudio 5 Real-Time PCR System, ThermoFisher Scientific, Walham, MA USA 02451) was performed on every blood sample used to prepare smears, to confirm the microscopic species identification. Using the validators’ microscopy results and the PCR results a consensus malaria species identification was made. Detailed methodology and illustrative images are provided in Additional files 3 and 4, respectively.

In September 2018, in an effort to increase the stock of non-falciparum slides, staff travelled to a malaria-endemic town in Kenya. For a week, they prepared blood smears from malaria-positive blood samples collected at two malaria testing sites. Smears were prepared using the same process detailed above, except that after smears dried, slides were immediately packaged and airfreighted on ice to the NICD the following morning. Slides were received within 24 h of production and were stained within 2–4 days.

The cost of setting-up an MSB was calculated using 2020 cost estimates. The year 1 or initial costs included the once-off costs of equipment purchases and installation, as well as the cost of preparing 50 slide batches (of 100 slides each). The recurrent year-on-year costs of the MSB included the costs of equipment maintenance and preparation of 100 slide batches. The cost of two, 2-week field trips a year, to recruit patients and prepare smears, was included in the annual budget.

From this MSB, slide sets were assembled and shipped by airfreight to participating laboratories for microscopist training and for a proficiency testing scheme (PTS). Registration of laboratories in the PTS was first approved by WHO/E8. The PTS was designed using the guidelines provided in the WHO Malaria Microscopy QA Manual, Chapter 11 [7]. For both the training and PTS activities, slides were selected from the slide storage cabinets, re-labelled, packed into slide boxes, and shipped. A questionnaire was administered to PTS participants in December 2020 for monitoring and improvement purposes.

## Results

**Fig. 1:**
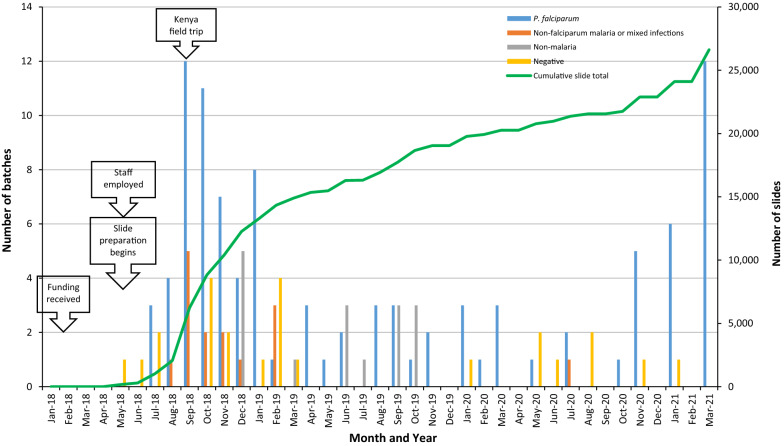
Timeline showing number of slide batches (bars) and cumulative number of slides (line) prepared for the NICD/E8 Malaria Slide Bank, January 2018–March 2021

Figure 1 is a summary of the 154 batches (26,623 slides) prepared from May 2018 to March 2021. The month with the highest number of slides made was September 2018, in which 14 batches were prepared in Kenya; this included five non-falciparum malaria and mixed species infections. There were 67 batches prepared in 2018, 44 in 2019, 24 in 2020 and 19 in 2021. The average batch size was 170 (range: 20–392). The majority of slide batches was *Plasmodium falciparum* (64%), followed by malaria-negative (16%), non-malaria (10%) and non-falciparum or mixed infections (10%). Approximately half of the batches were prepared from clinical samples diluted with donor blood.

Ninety-two percent (141/154) of batches passed internal QC. Of these, 104 (84%) were sent for validation to level 1 microscopists in Zambia, Botswana and Mozambique. Eighty-nine percent (93/104) passed this external validation and these batches were added to the MSB stock. Of these, 72 were malaria-positive batches which were all counted by the validators. The median count of the validators’ counts was used as the true count for each batch; true counts ranged between 48 and 240,486 p/µl. The true counts were assigned to arbitrary categories; 25% (18/72) were considered low counts (< 300 p/µl), 24% (17/72) medium counts (300–999 p/µl) and 51% (37/72) high counts (≥ 1,000 p/µl). PCR results were concordant with the validated microscopy results for all production batches. There were four samples that had an additional species-positive PCR result with a very high Ct value (indicating submicroscopic infections) that did not match microscopy results and these were excluded.

The initial cost of establishing an MSB was calculated at roughly $165,000, using the Rand/Dollar exchange rate on 30 June 2020 (17.28); see Additional file 5. The recurrent year-on-year costs were roughly $130,000, or $16,000 per E8 country. The main cost drivers in the recurrent costs were travel and staff. The following project-specific requirements were excluded from costing: (1) existing staff time, estimated as 0.20 × full time equivalent in the first year; (2) malaria microscopy training and ECAMM workshop attendance (E8 sponsored); (3) an air-conditioned room with benches, cupboards and shelves; (4) safety consumables such as PPE and waste containers, and (5) basic stationery.

From this slide bank, in 2019–2020, two training slide sets were sent to each country’s malaria reference laboratory, and five malaria microscopy proficiency testing scheme (PTS) surveys were sent to 12–14 participating laboratories. There was a 79% (11/14) response rate to the PTS participant feedback questionnaire. Of the laboratories that responded, the majority (73%, 8/11) did not participate in any other malaria microscopy PT/EQA. All 11 respondents were satisfied with the PT service and rated the quality of the blood films an average score of 4.5 out of 5. The online submission process was rated an average score of 4.4 out of 5, but some technical difficulties with the online system were noted. Suggestions for improvement were to start the programme earlier in the year and to include *Plasmodium vivax* slides.

## Discussion

This MSB is one of the key initiatives in the E8 Regional Malaria Diagnosis Programme that aims to provide regional support to help develop robust quality assurance programmes to improve the performance and quality of malaria diagnostic services provided within the region. Here, it was shown that with adequate funding, well-trained staff and clear protocols, setting up an MSB is an achievable task. In 35 months, 154 batches were prepared; although this was fewer than planned, the total number of slides prepared was on target. There were also far fewer batches of non-falciparum malaria smears than anticipated. A major contributing reason for the reduced number of batches, particularly non-falciparum smears, was the COVID-19 pandemic that resulted in a decrease in local malaria cases being identified, and the cancellation of the second field trip to a highly malaria-endemic country.

There were many lessons learned during the development of this MSB, which may be instructive for other groups. One of the limitations of the residual positive-blood sample acquisition process was that many blood samples were not received within 6 h of collection, but more usually within 24 h. While in principle, blood smears should be prepared as quickly as possible following venepuncture to ensure optimal parasite morphology and staining [7, 10], some older samples may be used successfully. To nevertheless maintain a high standard, the quality of the parasite and blood component morphology and staining was checked before mass smear preparation. The majority of samples remained acceptable within this time-frame. Of interest was that trypanosomes survived in EDTA-blood for a few days and, therefore, their morphology was maintained for longer than malaria parasites. Lastly, all MSB slides were independently validated by unbiased level 1-certified microscopists, which further confirms the acceptability of these smears. A second limitation of the sample collection procedure used, was that there was no control over the volume of blood received in the samples, hence the large range of batch sizes in this MSB. These blood samples were collected for diagnostic purposes and residual post-testing blood was used for the MSB. It is preferable to actively and immediately collect EDTA-blood and prepare smears on site from patients who test positive for malaria, as was done during the slide preparation in Kenya.

Smear uniformity within batches is very important for an MSB, as the slides should be interchangeable. The use of slide templates, measuring pipettes and regular blood sample mixing during slide preparation assists with homogeneity. Slide validation is a key component in the MSB process, as it provides multiple, independent, and unbiased expert reviews of the smears. The E8 sponsored the attendance of core microscopists from E8 countries in External Competence Assessment in Malaria Microscopy (ECAMM) workshops, and as a result the NICD were able to request, in return, the voluntary support of level 1 microscopists for slide validation. Initially, inadvertent collusion between microscopists was noted, and therefore not all the validation results were used. This issue was resolved with communication and revised instructions indicating that slide validation must be performed independently. PCR was most useful in cases of relapsing malaria species, where microscopic identification was often difficult. PCR results that indicated very scanty infections (high Ct values) that were not detected by microscopy were excluded from the composite diagnosis, as done by others [10].

When fixing blood films in methanol, it is best to use ‘fresh’ methanol. Due to the hydrophilic nature of this chemical, it becomes gradually diluted when exposed to air moisture, thereby losing its fixative efficacy. To overcome this, it was found that aliquotting methanol from a new container into smaller bottles kept the methanol effective. These bottles should be filled to capacity, kept tightly capped until used, and labelled with safety and lot details. When planning for smear preparation in Kenya, it became evident that it would be preferable to perform staining in South Africa. The concern was to prevent fixation of thick smears due to exposure to high humidity and heat before staining. To overcome this, smears were packed on ice and airfreighted to South Africa within 24 h of preparation. Smears were initially stained within 2 days of preparation but condensation on the slides from the shipping conditions (despite desiccant) resulted in this being extended to 3–4 days.

The biggest challenge experienced when developing the MSB was obtaining the less common malaria species; a field trip to a high-burden country was extremely beneficial in this regard. An alternative is to purchase slides from other, well-established slide banks. However, the quality of these slides will need to be thoroughly assessed before being accepted. To increase the diversity of the *P. falciparum* stock in the MSB, dilution to desired parasitaemias is very worthwhile. When diluting blood to obtain lower parasitaemias, it is best to use donor blood of the same ABO blood group to prevent erythrocyte clumping. The inclusion of some non-malaria parasites is good as microscopists should be aware of other pathogens that they may observe when performing malaria microscopy [11].

Based on the costing shown, the initial costs of setting-up an MSB are substantially reduced if an existing malaria laboratory with the basic equipment, consumables and reagents is used. The estimated minimum funding required for annual maintenance of the MSB is reasonable, especially as it is able to serve multiple countries in a region. The largest cost components are travel and staff salaries. The costs here were calculated on the quantity of the items needed to prepare 100 slide batches per year. However, many items are only sold in larger quantities, which will inflate the actual initial cost but also reduce recurrent year-on-year costs. Consistent internal quality control of smears pre- and post-staining avoids the unnecessary cost of shipping poor-quality batches for validation. As mentioned, the cost of slide validation was reduced due to the E8 sponsoring the level 1 microscopists attendance at the ECAMM workshops. There are other options to cut costs, for example the use of bottled drinking water to dilute the Giemsa stain [12], or the use of a cheaper molecular assay for species confirmation.

As well as producing slides for microscopist training, an MSB can supply proficiency testing schemes. There is a demand for malaria microscopy PT schemes that comprehensively assess species identification and parasite quantitation. The overall good feedback from participants endorses the quality of this MSB and PTS. In addition to supplying slides for training and PTS, an MSB may loan or sell slides. It is the intention of this MSB to offer these services, but it is dependent on the growth of the slide bank. Additionally, if this MSB grows substantially the slides and the NICD/E8 Malaria Microscopy PT Scheme has the potential to support countries outside of the E8. There has been a large number of requests to join this PTS from health institutions in countries within the E8, such as Botswana, Zimbabwe and Eswatini, and from outside the region, such as The Gambia, Kenya, Tanzania and Rwanda.

An excellent MSB has first-rate, validated smears i.e., uniform macroscopic and microscopic appearance and good quality staining. There are very few published accounts of MSBs [10, 13]. However, from personal communications, there are some countries that have started their own MSBs. The limitations of these MSBs may include varying quality, limited species, and un-validated smears that may mislead microscopists. It is not feasible for every country to develop its own MSB, especially due to lack of resources and more importantly, limited access to positive blood samples. For this reason, it is preferable to develop a regional slide bank as described here that can support multiple countries.

## Conclusions

Malaria specimen banks in general are in demand, especially in Africa. Such banks can provide various species and strains of malaria in different forms including microscopy slides, dried blood spots, RDT controls and parasite DNA. This MSB has proven to be an essential tool in the region as it moves towards malaria elimination. The E8 participating laboratories have become dependent on this MSB especially as for many of them, it is the only source of PTS material. Unfortunately, the grant funding for this project was limited and unless further funding is acquired, this valuable resource will cease to exist.

## Supplementary Information

**Additional file 1.** Overview of training programme for new MSB staff.

**Additional file 2.** List of laboratories used for MSB sample collection.

**Additional file 3.** Detailed procedure for mass slide production.

**Additional file 4.** Images highlighting steps in the slide preparation and staining process. One composite image made of six individual images.

**Additional file 5.** Costing for MSB set-up and annual MSB maintenance.

## Data Availability

The datasets used and/or analysed during the current study are available from the corresponding author on reasonable request.

## Abbreviations

AFRO: Regional Office for Africa
Ct: Cycle threshold
DNA: Deoxyribonucleic acid
E8: Elimination Eight
EDTA: Ethylenediaminetetraacetic acid
ECAMM: External competence assessment in malaria microscopy
EQA: External quality assessment
MSB: Malaria slide bank
NICD: National Institute for Communicable Diseases
PCR: Polymerase chain reaction
PTS: Proficiency testing scheme
QC: Quality control
RDT: Rapid diagnostic test
RITM: Research Institute for Tropical Medicine
SADC: Southern Africa Development Community
WHO: World Health Organization
