# Supplementary material for: Improving the quality of malaria diagnosis in southern Africa through the development of a regional malaria slide bank
Source: Malar J. 2021 Sep 8;20:365. doi: 10.1186/s12936-021-03899-5 (PMC8424146; doi:10.1186/s12936-021-03899-5)
Supplement: Supplementary file 1 — Additional file 1. Overview of training programme for new MSB staff. [file 12936_2021_3899_MOESM1_ESM.docx]

**Additional file 1**. Overview of training programme for new MSB staff

1. **Parasitology Laboratory at the National Institute for Communicable Diseases** (Internal training)

**Summary:**

Staff were trained on different components as listed below, theoretical information and/or Standard Operating Procedures (SOPs) were provided. For practical activities, demonstrations were done, followed by supervised hands-on practice and lastly an assessment of competency.

**Programme:**

| **Training component** | **Topic/Activity** |
| --- | --- |
| Safety | General laboratory safety including OSH Act requirements |
|  | Parasitology NICD-laboratory specific training |
| Quality assurance (QA) | General QA and ISO 15189 requirements |
| Blood parasite methods | Blood film preparation and staining for malaria and other blood parasites |
|  | Malaria rapid diagnostic tests |
|  | Trypanosome and microfilaria concentration methods |
| Equipment | Microscope usage and maintenance |
|  | pH meter usage and maintenance |
|  | Coverslipper usage and maintenance |
| Microscopy | Malaria (*Plasmodium* species) parasite identification |
|  | Other blood parasite identification |
|  | Malaria parasite quantitation |
| Malaria slide bank methods | Mass blood film preparation, Giemsa staining and quality control |
|  | Labeling and packaging |
|  | Packing and shipping (in line with IATA regulations) |
|  | Inventory database usage and maintenance |
| Proficiency testing (PT) | Management of the PT scheme |
|  | Online database for resulting and reporting |

**B. WHO Malaria Slide Bank Workshop** (External training)

**Aim:**

The objective was to train laboratory personnel from national malaria programmes and research institutions on how to develop, run and maintain malaria slide banks (MSBs) using WHO recommended Standard Operating Procedures (SOPs).

**Agenda:**

| **Day** | **Topic/Activity** |
| --- | --- |
| Days 1 and 2 | Lecture, discussion and practical/hands-on training on the SOPs and forms used in slide banking |
| Day 3 | Practical session on donor screening and selection, venous blood sample collection and batch blood film preparation |
| Day 4 | Practical session on batch staining |
| Day 5 | Lectures/presentations on MSB SOPs  Development of country work plans and way forward |

1. **WHO External Competence Assessment in Malaria Microscopy** (External certification)

**Aim:**

The objective was to assess microscopists on the ability to diagnose malaria.

**Agenda:**

| **Day** | **Topic/Activity** |
| --- | --- |
| Day 1 | Pre-course theory test – 25 general malaria microscopy questions.  Pre-course practical test – 18 slides, species detection, identification and counts.  *These do not count towards the final grading.* |
| Day 2-4 | Mornings: presentations (primarily a review) of all aspects of malaria microscopic diagnosis, from specimen collection to diagnostic reporting.  Plus a review of previous days slides.  Afternoons: 56 test slides for assessment and grading. |
| Day 5 | Final results and review. |
