# Supplementary material for: Improving the quality of malaria diagnosis in southern Africa through the development of a regional malaria slide bank
Source: Malar J. 2021 Sep 8;20:365. doi: 10.1186/s12936-021-03899-5 (PMC8424146; doi:10.1186/s12936-021-03899-5)
Supplement: Supplementary file 2 — Additional file 2. List of laboratories used for MSB sample collection. [file 12936_2021_3899_MOESM2_ESM.docx]

**Additional file 2**. Laboratories used for MSB sample collection

1. Chris Hani Baragwanath Laboratory, National Health Laboratory Service, Soweto, Johannesburg
2. Charlotte Maxeke Johannesburg Academic Hospital Laboratory, National Health Laboratory Service, Parktown, Johannesburg
3. Helen Joseph Hospital Laboratory, National Health Laboratory Service, Auckland Park, Johannesburg
4. Tambo Memorial Hospital laboratory, National Health Laboratory Service, Boksburg, Johannesburg
5. Tembisa Hospital Laboratory, National Health Laboratory Service, Tembisa, Johannesburg
6. Cecilia Makiwane Hospital Laboratory, National Health Laboratory Service, Mdantsane, East London
7. Ampath Laboratories, Centurion, Pretoria
8. Vermaak & Partners Laboratory, Rosebank, Johannesburg
