# Supplementary material for: Improving the quality of malaria diagnosis in southern Africa through the development of a regional malaria slide bank
Source: Malar J. 2021 Sep 8;20:365. doi: 10.1186/s12936-021-03899-5 (PMC8424146; doi:10.1186/s12936-021-03899-5)
Supplement: Supplementary file 3 — Additional file 3. Detailed procedure for mass slide production. [file 12936_2021_3899_MOESM3_ESM.docx]

**Additional file 3**. Detailed procedure for mass slide production

For mass smear preparation, slides were placed on slide templates for one thick and one thin blood film to be made on the same slide (see image on Additional file 4 – a). Two staff members worked in tandem, one dispensing blood and the other immediately making the smear. All the thin films were made first as they dry very quickly unlike thick films which can easily be smudged. Measuring pipettes were used to dispense 2 µl for each thin blood film and 6 µl for each thick blood film; blood was gently mixed regularly with a plastic Pasteur pipette to ensure homogeneity. Thin films were spread to ensure good feathered edges and thick films were circularly and evenly spread to a diameter of 12 mm, without any gaps, as per the WHO Basic Malaria Microscopy Learner’s Guide [1]. Poorly prepared smears were discarded. Desired parasite densities (parasitaemias) were prepared by diluting positive blood with healthy donor blood of the same blood group.

Slides were left to dry for at least one day, after which the thin blood films were fixed with 100% methanol (Merck KGaA, 64271 Darmstadt, Germany). The end of the slide with the thin blood film was immersed in a 25 ml beaker of methanol for <5 seconds, and the excess methanol was drained off on paper towel (Additional file 4 – b). Care was taken to ensure that the methanol or methanol fumes did not come into contact with the thick blood films. Slides were mass-stained 2-4 days after smear preparation. Slides were packed into slide-staining holders (100-place plastic slide boxes with the bases cut out), either individually (staining 100 slides) or back to back (staining 200 slides). The holders with slides were placed into 1.5 L flat rectangular plastic staining containers. Separate holders and containers were used for negative smears. At least four slides were marked as quality control (QC) slides and were strategically positioned in the staining holder to monitor the staining process. A 3% Giemsa stain solution was prepared by adding Giemsa (Merck KGaA, 64271 Darmstadt, Germany) to phosphate buffer of pH 7.2 (Diagnostic Media Products, Johannesburg, South Africa). The pH of the buffer was checked before each use with a pH meter (Orion Star Benchtop pH Meter, ThermoFisher Scientific, Walham, MA USA 02451), and adjusted if necessary (Additional file 4 – c). The stain solution was mixed well and gently poured into the staining containers until the slides were completely submerged in stain (Additional file 4 – d). Slides were left to stain for 30 minutes, after which they were washed with tap water (~pH 7.2), as follows. The end of a rubber tube that was attached to the tap was inserted into the corner of the staining container; water was allowed to slowly wash the stain off the slides (Additional file 4 – e). The iridescent stain scum floated off first and slides were rinsed in this way until the water ran clear. The slide staining holders with slides were removed and placed on paper towels to drain the excess water. Slides were then individually placed in wooden drying racks and allowed to dry in a vertical position (Additional file 4 - f).

After drying, slides underwent internal QC whereby all slides were checked macroscopically and the four QC slides were microscopically examined. Poor quality slides, such as ones with substantial wash-off, clumping of parasites/white blood cells or excessive stain precipitate, failed QC and were discarded. See Additional file 4 – g for a well-prepared and well-stained thick and thin blood smear. Quality control slides of positive batches were also thoroughly examined to establish a final microscopic identification and parasite count, using methods detailed below.

For low parasitaemias (generally <1%/50 000 p/µl), parasite counts were performed on thick blood films, as per WHO methods [1]. For higher parasitaemias, parasite counts were performed on thin blood films as follows. In the monolayer of the thin blood film, the number of red blood cells (RBCs) infected with asexual parasites and the total number of red blood cells were counted per high power field (HPF). For parasitaemias with ≤2 infected RBCs/HPF, a minimum of 4 000 total RBCs was counted, and for higher parasitaemias, a minimum of 2 000 total RBCs was counted. The percentage infection was calculated by dividing the total number of infected RBCs by the total number of RBCs counted, and multiplying by 100.

Slides from batches that passed QC were sent to level 1 microscopists within the E8 countries, for slide validation. Two slides from each batch were read blinded by at least six microscopists, providing 12 independent results. Results included a microscopic identification, and if malaria positive a parasite count as well. The median count of all the validators’ counts was used as the true count for each batch. The true counts were assigned to arbitrary categories: low counts (<300 p/µl), medium (300-999 p/µl) and high counts (≥1 000 p/µl). Batches that passed validation were added to the MSB inventory, and those that failed were discarded.

A real-time PCR assay using a commercial kit (RealStar Malaria Screen & Type PCR Kit 1.0, Altona Diagnostics, Hamburg, Germany; QuantStudio 5 Real-Time PCR System, ThermoFisher Scientific, Walham, MA USA 02451) was performed on every blood sample used to prepare smears, to confirm microscopic result. Using the validators’ microscopy reports and the PCR results, a consensus malaria species identification was made. For long-term storage, slides were labelled with the batch number, and coverslipped using an automated coverslipping instrument (Leica Biosystems, Buffalo Grove, IL 60089, United States). After drying, slides were stored in labelled slide cabinets at room temperature.

**Reference**

1. World Health Organization. Basic Malaria Microscopy Part I. Learner’s guide. 2nd ed. 2010.
