# Supplementary material for: Improving the quality of malaria diagnosis in southern Africa through the development of a regional malaria slide bank
Source: Malar J. 2021 Sep 8;20:365. doi: 10.1186/s12936-021-03899-5 (PMC8424146; doi:10.1186/s12936-021-03899-5)

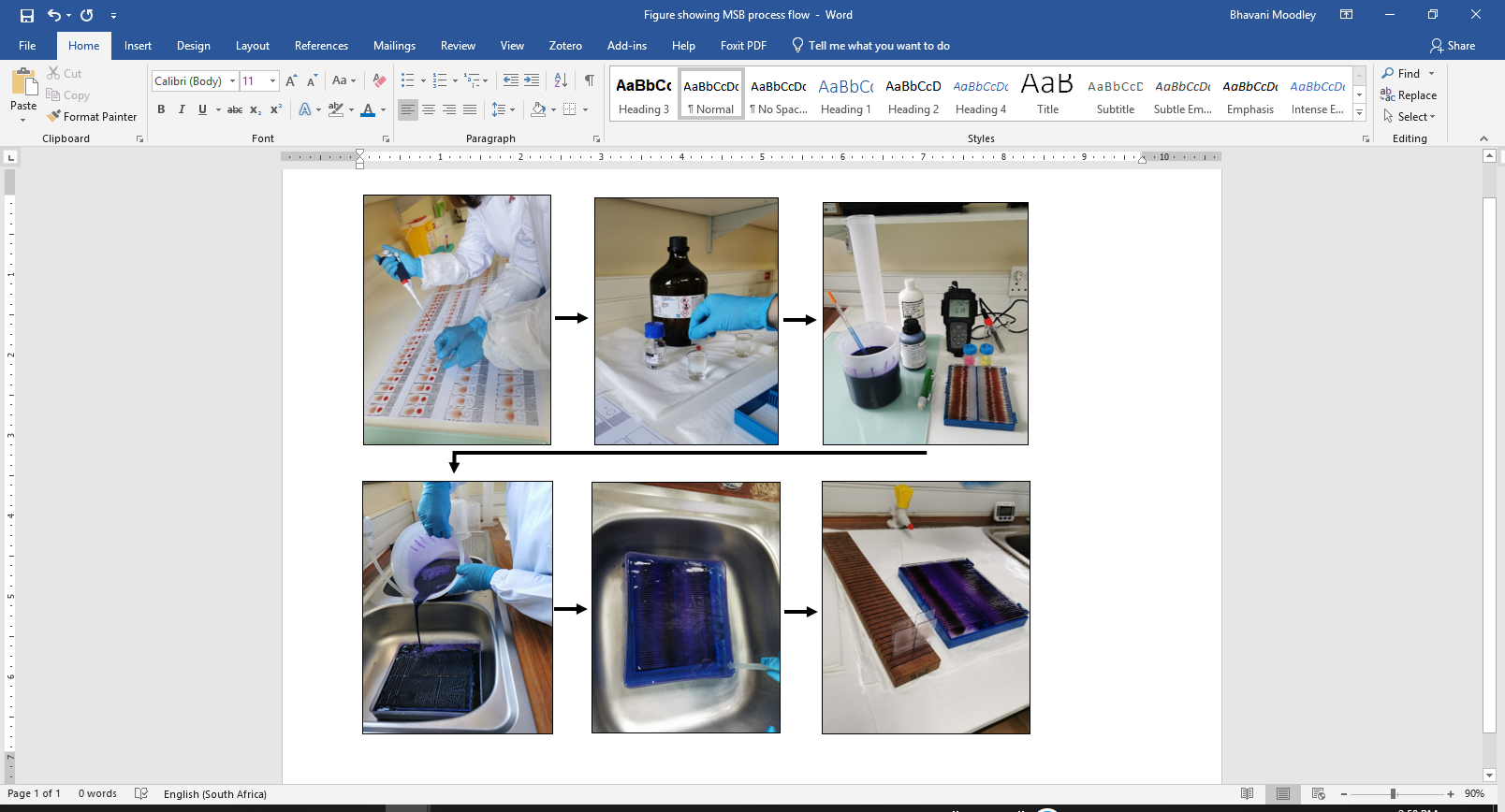
**Additional file 4**. Images highlighting steps in the slide preparation and staining process. **a** – mass thick and thin blood film preparation using slide templates and measuring pipettes; **b** – thin blood film fixing in 100% methanol; **c** – buffer pH checking using pH meter and calibration reagents; **d** – mass smear staining with 3% Giemsa stain; **e** – gentle washing of smears by flooding container with running water; **f** – smear drying; and **g** – a well-stained slide.


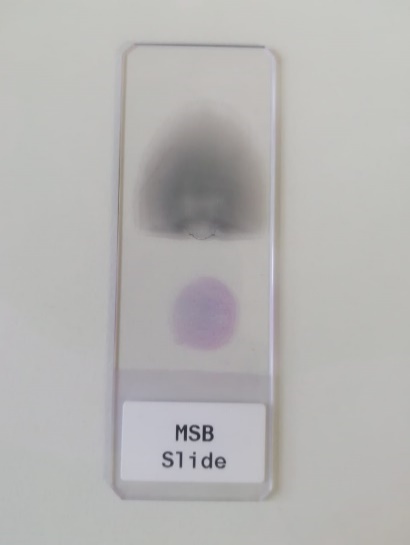


**g**

**a.**

**c.**

**b.**

**d.**

**e.**

**f.**

**
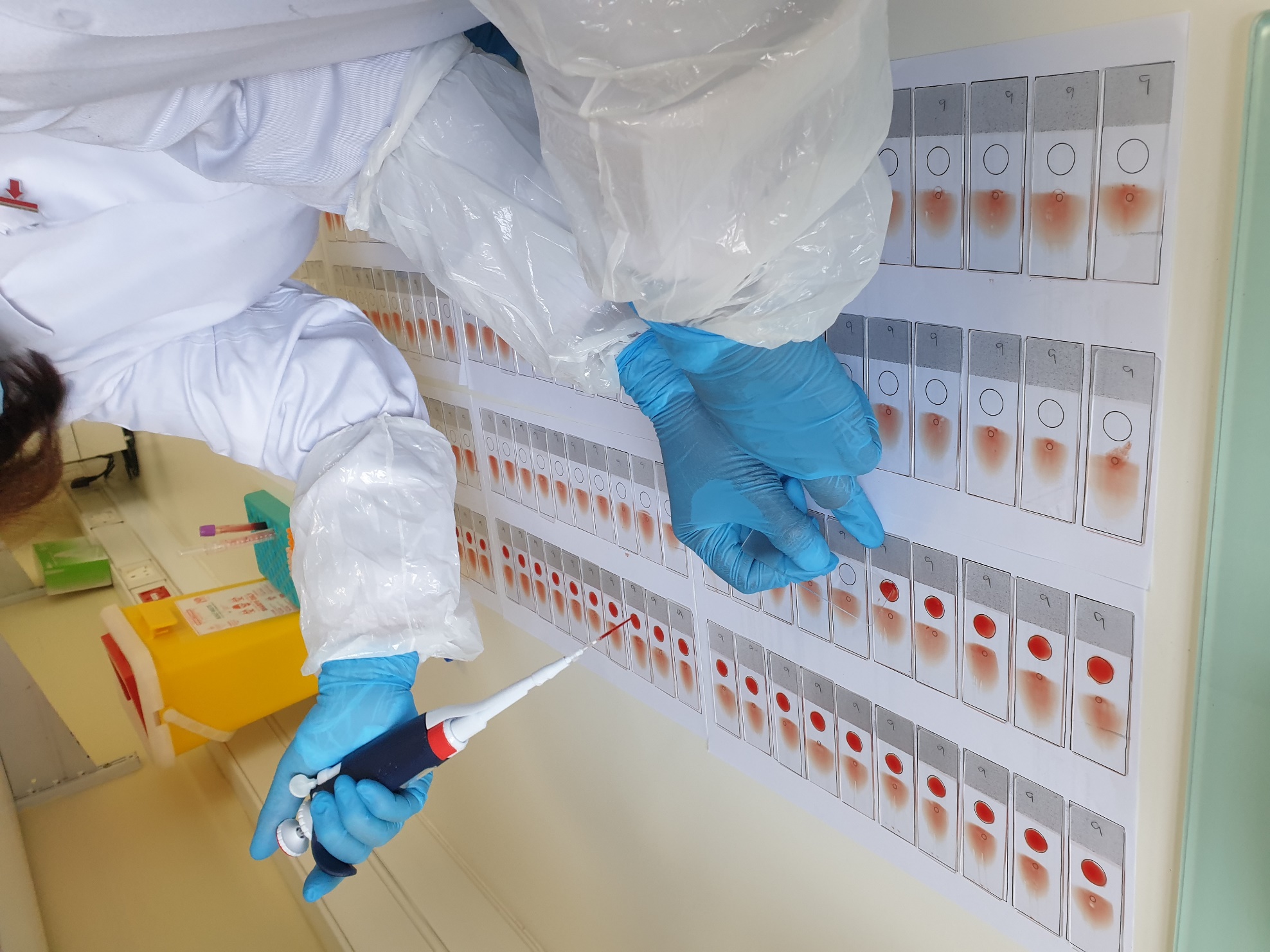
**


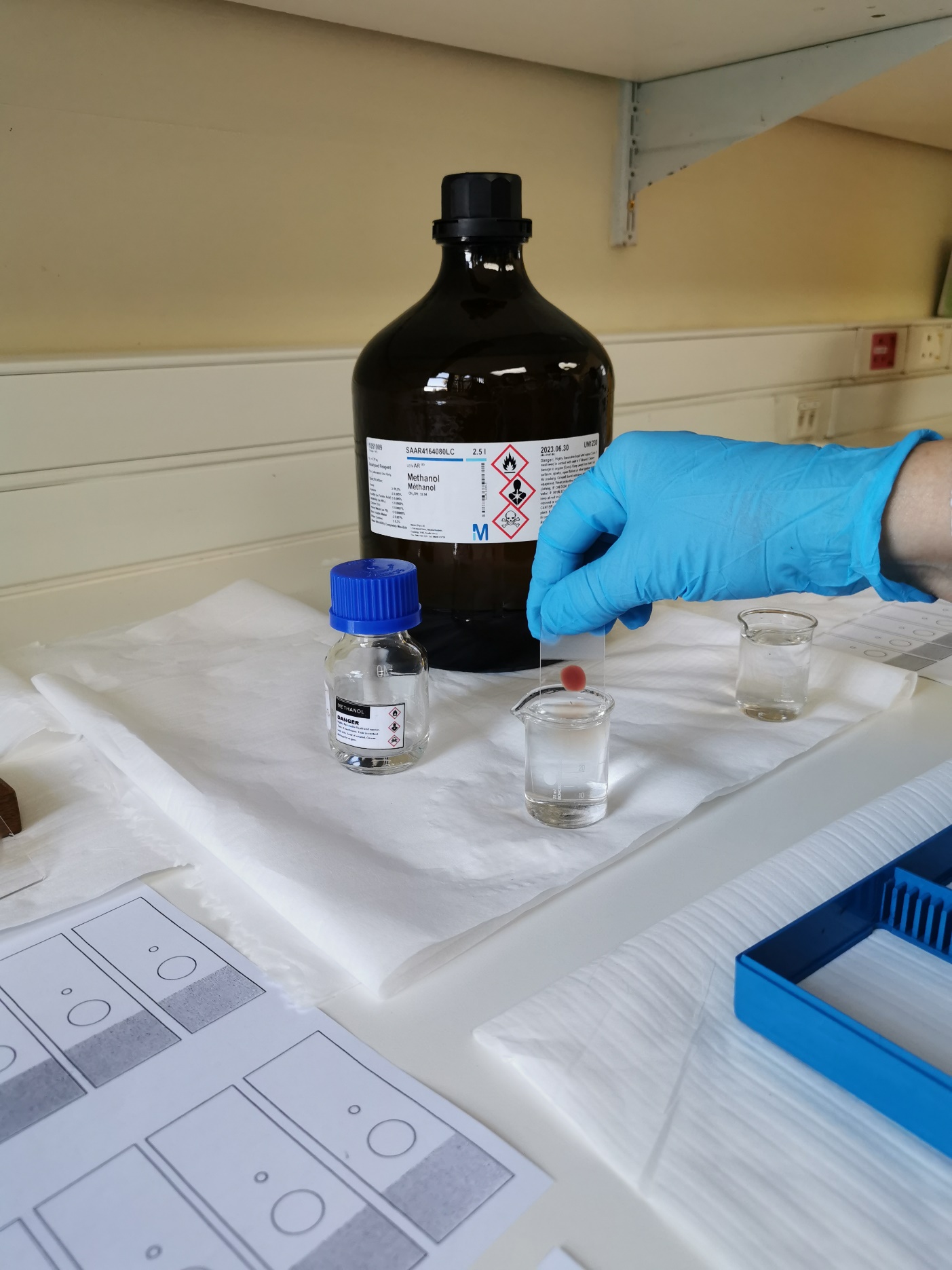


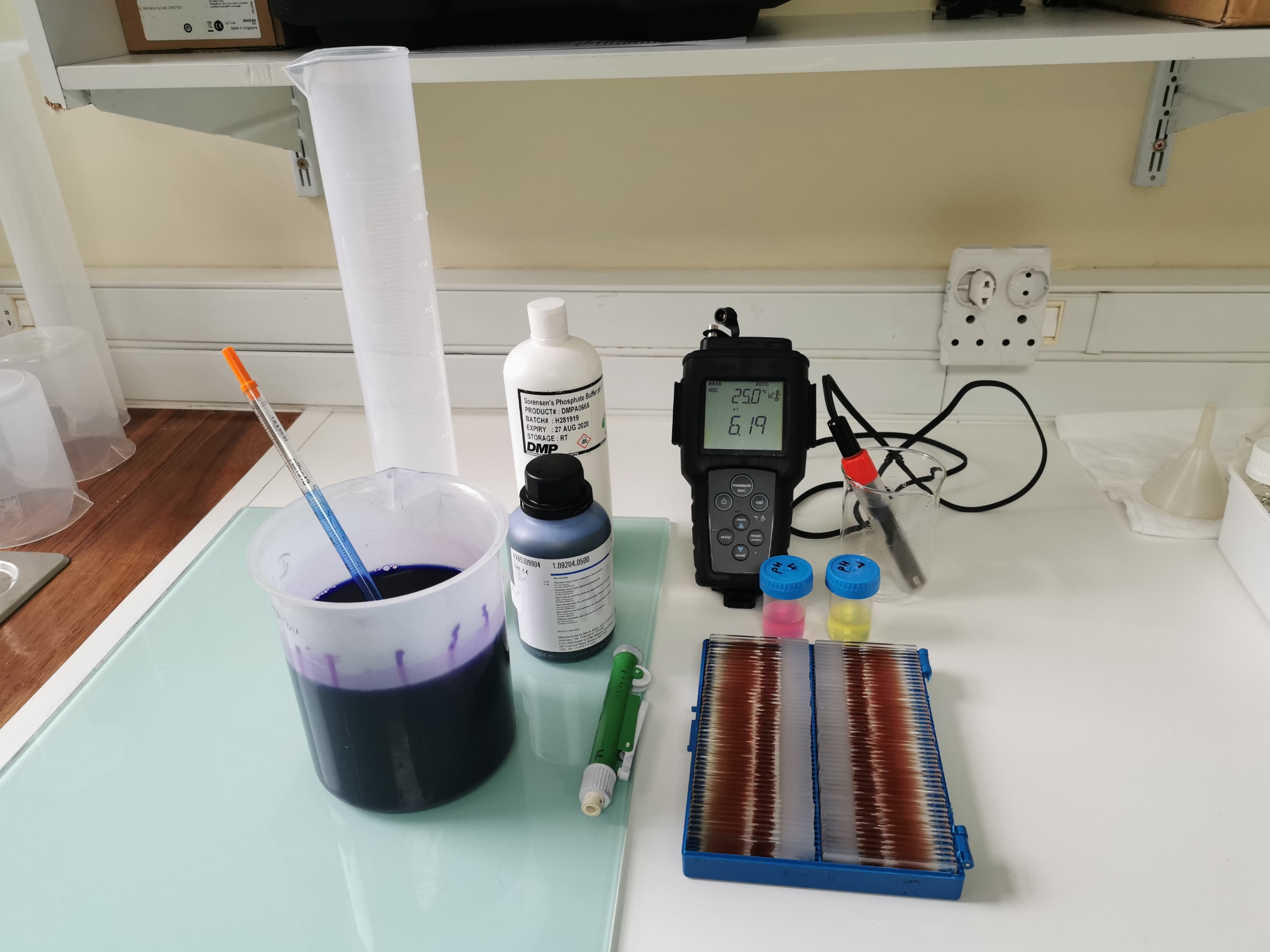


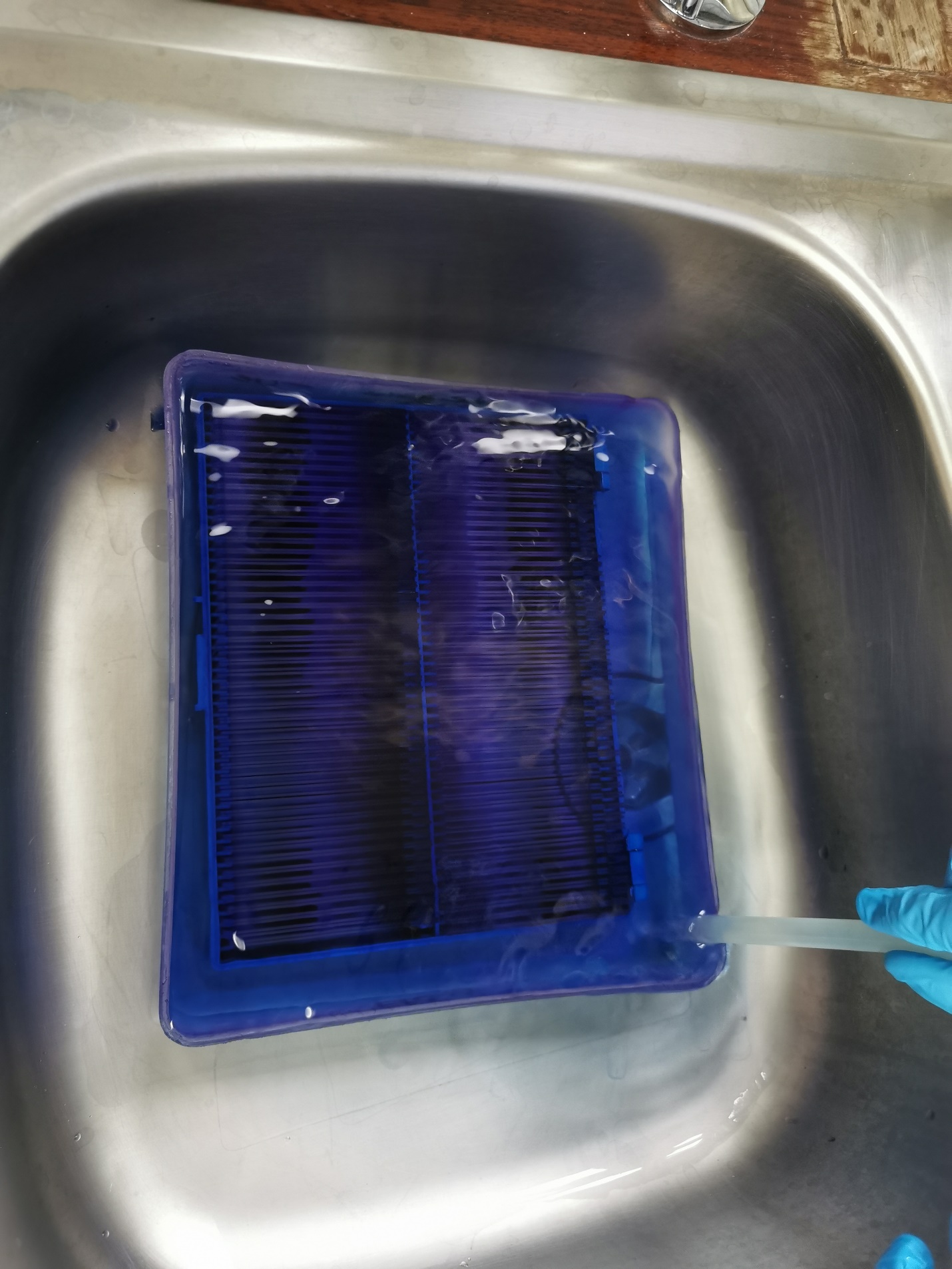


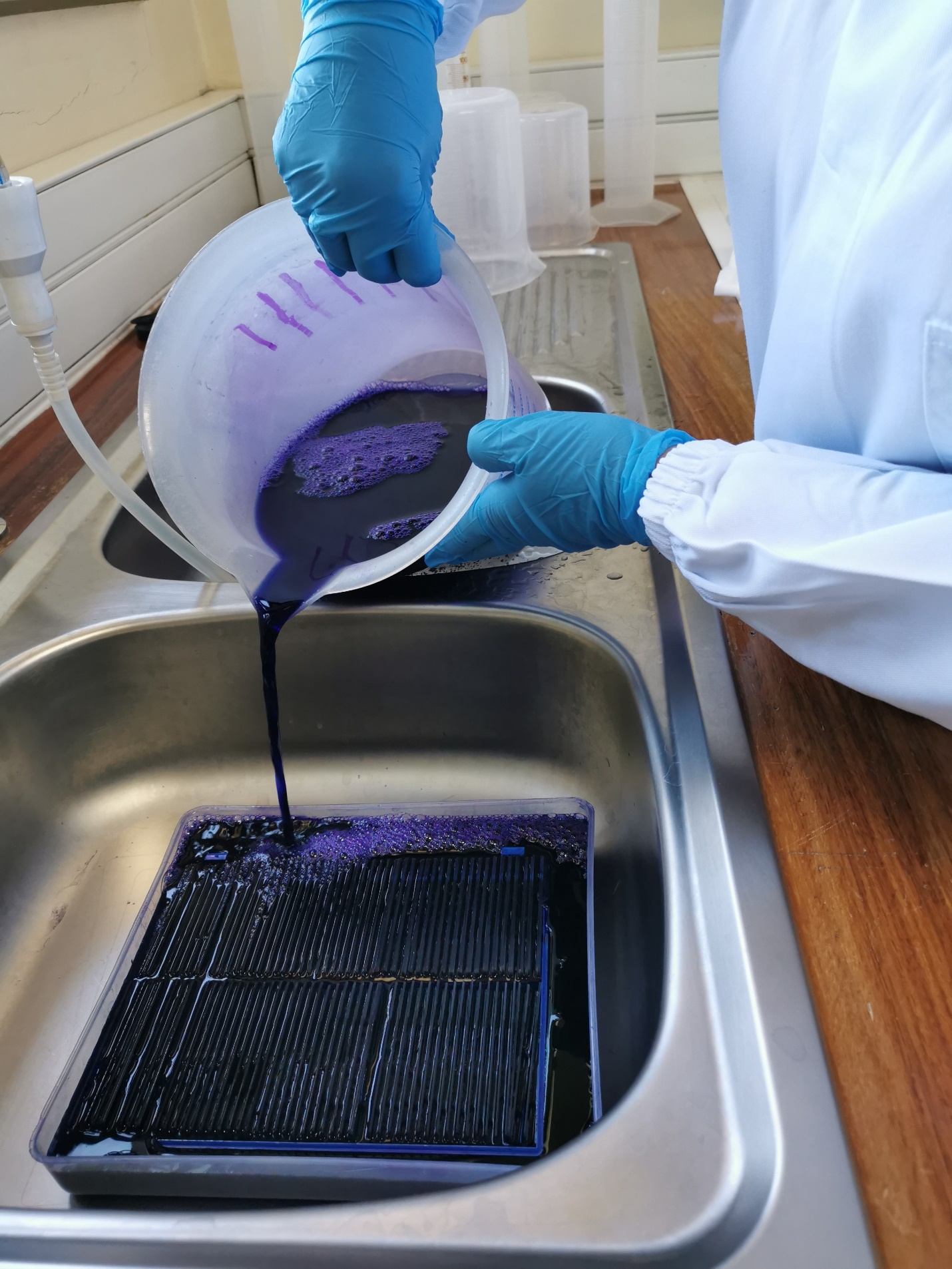


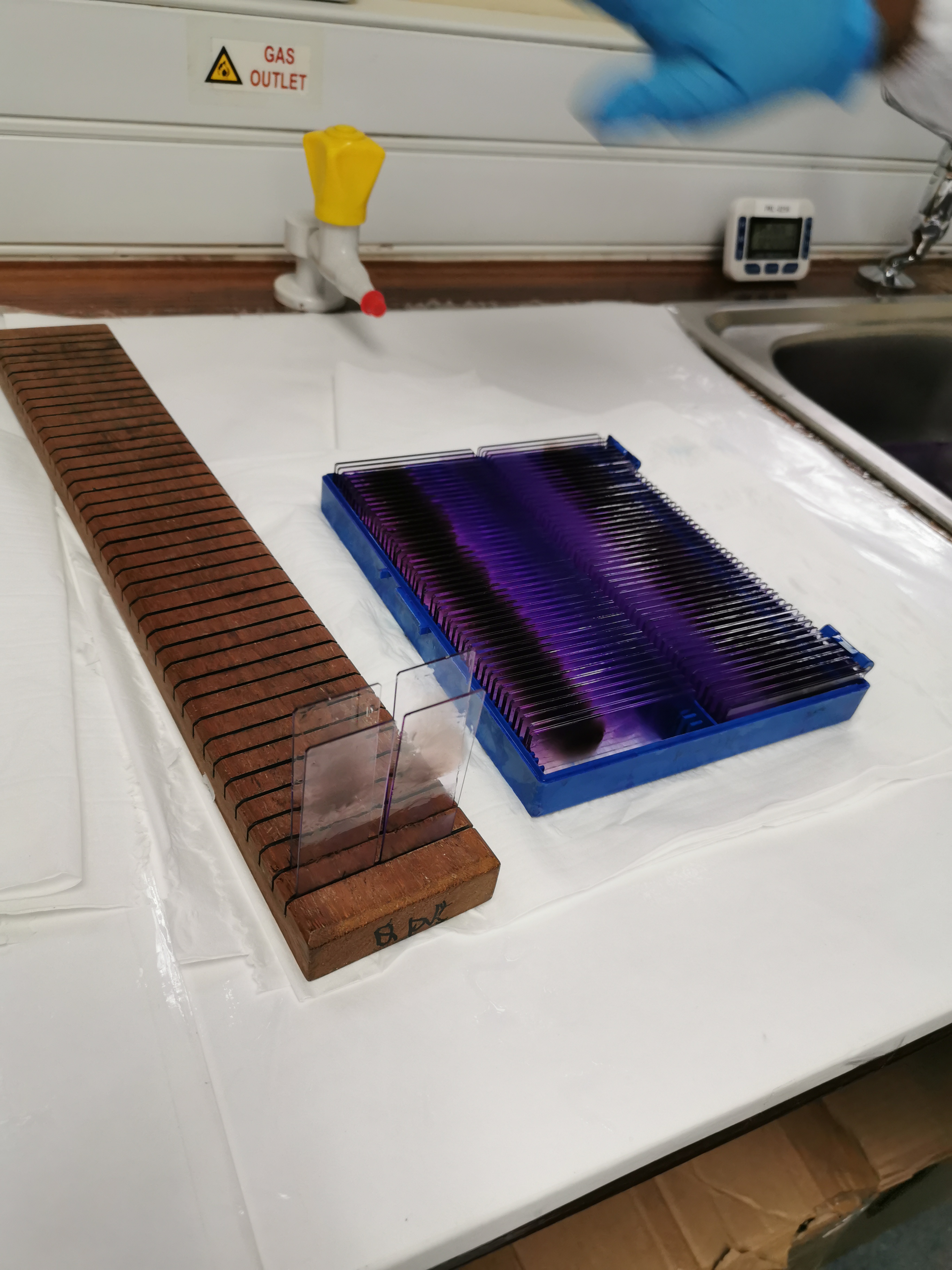


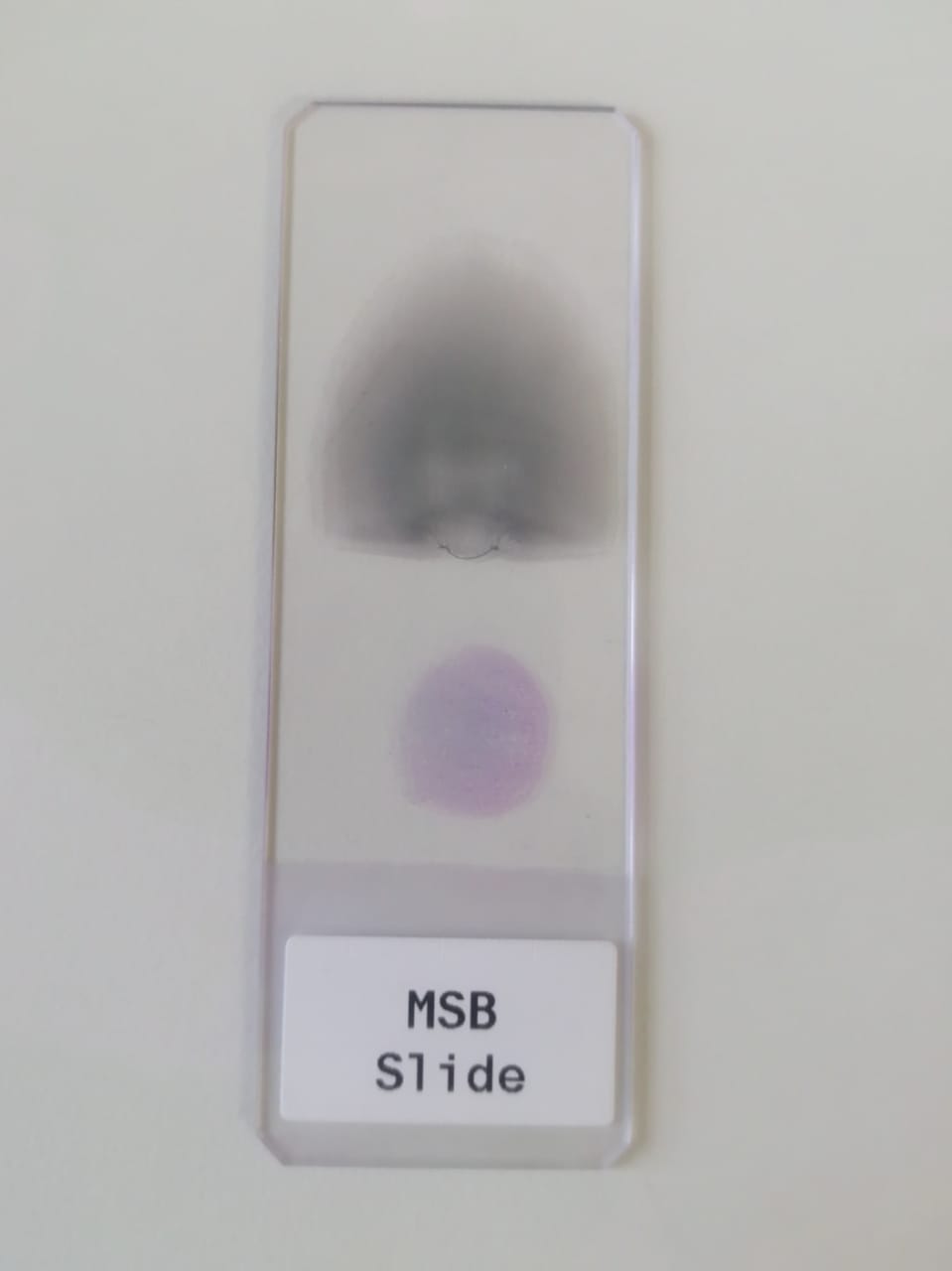

Supplement: Supplementary file 4 — Additional file 4. Images highlighting steps in the slide preparation and staining process. One composite image made of six individual images. [file 12936_2021_3899_MOESM4_ESM.docx]
